# Mapping the organizational readiness to change assessment to the Consolidated Framework for Implementation Research

**DOI:** 10.1186/s43058-021-00121-0

**Published:** 2021-02-13

**Authors:** Jennifer Kononowech, Hildi Hagedorn, Carmen Hall, Christian D. Helfrich, Anne C. Lambert-Kerzner, Susan C. Miller, Anne E. Sales, Laura Damschroder

**Affiliations:** 1 Center for Clinical Management Research, VA Ann Arbor Healthcare System, 2800 Plymouth Rd, Ann Arbor, MI USA; 2 Center for Care Delivery and Outcomes Research, Minneapolis VA Health Care System, 1 Veterans Drive, Minneapolis, MN USA; 3 Department of Psychiatry, University of Minnesota School of Medicine, 606 24th Ave S, Minneapolis, MN USA; 4 Gusek Hall Consulting, 1362 Ryan Ave. W Ste 101, Roseville, MN USA; 5 Seattle-Denver Center of Innovation, VA Puget Sound Health Care System, 1660 S. Columbian Way, Seattle, WA USA; 6 School of Public Health, University of Washington, 1959 NE Pacific St, Seattle, WA USA; 7 Department of Surgery, University of Colorado Anschutz Medical Campus, 12631 East 17th Avenue, Aurora, CO USA; 8 Department of Health Services, Policy & Practice, Brown University School of Public Health, 121 S. Main Street, Providence, RI USA; 9 Department of Learning Health Sciences, University of Michigan Medical School, 300 N. Ingalls Street, Ann Arbor, MI USA

**Keywords:** Implementation research frameworks, Surveys, Measurement, PARiHS, CFIR, ORCA

## Abstract

**Background:**

Implementation researchers recognize the influential role of organizational factors and, thus, seek to assess these factors using quantitative measurement instruments. However, researchers are hindered by instruments that measure similar constructs but rely on different nomenclature and/or definitions. The Consolidated Framework for Implementation Research (CFIR) provides a taxonomy of constructs derived from prior frameworks and empirical studies of implementation-related constructs. The CFIR includes constructs based on the original Promoting Action on Research Implementation in Health Services (PARiHS) framework which highlights the key roles of strength of evidence for a specific evidence-based intervention (EBI), favorability of organizational context for change, and capacities to facilitate implementation of the EBI. Although the CFIR is among the most frequently used implementation frameworks, it does not include quantitative measures. The Organizational Resource and Context Assessment (ORCA) is a quantitative measurement instrument that was developed based on PARiHS, assessing its three domains. Factors within these three domains are conceptually similar to constructs in the CFIR but do not match directly. The aim of this work was to map ORCA survey items to CFIR constructs to enable direct comparisons and syntheses of findings across studies using the CFIR and/or ORCA.

**Methods:**

Two distinct, independent research teams, each used rigorous constant comparative techniques with deliberation and consensus to map individual items from the ORCA to the five domains and 39 constructs of CFIR.

**Results:**

ORCA items were mapped primarily to three of five CFIR domains: Inner Setting, Process, and Intervention Characteristics. The two research teams agreed on 88% of mappings at the higher domain level; at the lower construct level, their mappings aligned for 62.2% of the ORCA items.

**Conclusions:**

Mapping results reveal that the ORCA focuses measurement prominently on Inner Setting, Process, and Intervention Characteristics. This mapping guide can help improve consistency in measurement and reporting, enabling more efficient comparison and synthesis of findings that use either the ORCA instrument or the CFIR framework. The guide helps advance implementation science utilizing mixed methods by providing CFIR users with quantitative measures for selected constructs and enables ORCA users to map their findings to CFIR constructs.

**Supplementary Information:**

The online version contains supplementary material available at 10.1186/s43058-021-00121-0.

Contributions to the literature

- First published mapping between two tools that are widely used in the implementation research literature.
- Researchers often feel an imperative to innovate, resulting in a burgeoning number of frameworks and instruments. This paper provides early work that maps a published instrument to an already widely used framework.
- This mapping guide can enable researchers to build broader evidence more efficiently about the key role of context across implementation research projects.

## Background

Implementation scientists have developed a number of instruments and frameworks to guide the implementation of evidence-based interventions (EBI) in real-world settings [1]. The Consolidated Framework for Implementation Research (CFIR) is a determinants framework that provides a menu of constructs or determinants (potential barriers and facilitators to implementation) by which to assess and describe context [2, 3]. It is among the most widely used implementation frameworks, which makes it a useful tool for reporting and referencing findings across studies [4]. The Organizational Resource and Context Assessment (ORCA) was developed based on the Promoting Action on Research Implementation in Health Services (PARiHS) framework and assesses PARiHS’ three key domains: strength of evidence for a specific EBI, the favorability of the organizational context for change, and capacities to facilitate the implementation of the EBI. Measurement is based on individuals’ perceptions of factors within these domains [5, 6]. The CFIR includes constructs based on the original PARiHS, and thus, items within the three domains assessed by the ORCA are conceptually similar to constructs in the CFIR, but do not match directly. Because of similarities, the subscales and individual items of the ORCA could potentially be mapped to CFIR constructs.

Implementation researchers need guidance about how constructs may overlap or link to one another across multiple instruments and frameworks to promote synthesis of findings across studies. In theory, measures could be mapped back to a common set of constructs, catalogued in one or more determinants frameworks [2]. Such a mapping would serve a practical purpose by providing a repository of quantitative measures (ORCA) linked to qualitative constructs (CFIR) that can be used in mixed methods research. Previous work has identified five major reasons for using mixed methods in implementation work including to use quantitative methods to measure implementation outcomes and qualitative methods to understand process, to conduct both exploratory and confirmatory research, to examine intervention content and context, to incorporate the perspective of consumers of EBIs, and to compensate for one set of methods by the use of another set of methods [7].

Mapping specific items within a measurement tool, such as the ORCA, to a framework that defines conceptual constructs that are important to implementation research, such as the CFIR, would facilitate more efficient comparison and synthesis of findings across a broader array of studies. Therefore, the purpose of this work was to map the items from the ORCA instrument to CFIR constructs.

### Brief overview of the CFIR

The CFIR is a meta-theoretical framework and provides a foundational taxonomy that sought to provide a uniform and defined set of conceptual constructs by which to study the role of context within implementation [3]. The CFIR provides a menu of constructs along with published approaches for using the framework to assess implementation determinants [8–10]. The CFIR is widely used; as of February 2020, there were nearly 1700 unique articles in PubMed, citing the original CFIR paper [11].

The CFIR constructs were derived from 19 different published theories, models, and frameworks and are organized in five domains: Intervention characteristics, Outer setting, Inner setting, Characteristics of individuals, and Process. The five domains describe 39 underlying constructs and sub-constructs related to each of the domains under which they are nested.

### Brief overview of the ORCA

The ORCA is a self-report, structured survey instrument to assess evidence and organizational-level perceptions, posited to influence the implementation of a specific EBI (or discrete set of EBIs). It has been used as part of several evidence-based practice implementation efforts in the Veterans Health Administration and elsewhere (e.g., [12, 13]).

The PARIHS framework, on which the ORCA is based, presents a model focused on a multi-level approach (intervention and organization) to implementing EBIs [5]. The ORCA operationalizes constructs in the PARIHS framework and consists of three scales:

- *Evidence*—the nature and strength of the evidence for the proposed change/innovation;
- *Context*—the quality of the organizational context to support change; and
- *Facilitation*—the organizational capacity to help people change their attitudes, behaviors, skills, and ways of thinking and working to facilitate the proposed change/innovation [4].

Understanding an organization’s perception of the evidence supporting the proposed change/innovation, available resources and context, and capacity to facilitate change will help determine if the organization is ready to implement the EBI [14]. By conducting a readiness assessment, organizations can identify determinants (barriers and facilitators) that can be used to prioritize sites for implementation, guide choices of implementation strategies to improve the likelihood of effective implementation, and/or be measured over time to assess the effectiveness of implementation strategies [15].

## Methods

Two distinct and independent research teams completed separate iterative mappings of the ORCA survey to the CFIR framework (CGH, CDH, LJD, HH, and ALK in research team 1 (RT1) and JK, SCM, and AES in RT2). Both teams began their respective process with review of the seminal papers related to the ORCA and CFIR and with pre-existing in-depth knowledge from application and included creators of the ORCA (CDH and AES) and CFIR (LJD). The parallel efforts by the teams were unintentional and were discovered when RT2 consulted with a team member from RT1. The teams agreed that combining efforts would strengthen the intention and impact of the work.

RT1 used an iterative analytical strategy, drawing on constant comparative methodology involving moving back and forth between the ORCA survey and the CFIR framework to support the evolution of the mapping process [16, 17]. Five implementation researchers (CGH, CDH, LD, HH, ALK) provided consensus leading to final results. This in-depth process occurred over a 1-year period between 2013 and 2014, meeting weekly to bi-weekly to establish clarity of the shared meaning of each construct with deliberation and consensus to code individual items from the ORCA based on definitions for the five domains and 39 constructs of CFIR. The rationale for each coding decision was documented.

Each of the ORCA items was reviewed by each reviewer, with its full stem (e.g., I.2a: The {Proposed practice changes or guideline implementation}…are (is) supported by randomized control trials (RCTs) or other scientific evidence…). Three reviewers (LJD, CGH, ALK) independently compared the ORCA item with each CFIR sub-construct and selected the CFIR sub-construct that most closely matched the content of the ORCA questionnaire item. The reviewers referred to published definitions of CFIR constructs and could make secondary selections (i.e., an additional CFIR sub-construct that appeared to match the item). A consolidated document was created to display coding by each researcher for each ORCA item. Items were color-coded based on whether the three sets were in total agreement (all three researchers chose the same construct), there was agreement by two researchers, or had no agreement.

Results were discussed with a larger group in a series of meetings until consensus was achieved (LJD, CGH, ALK, CDH), with notes recorded about the rationale for the final decision and alternatives that were considered. Later in the process, a fifth independent reviewer (HH) was added to ensure objectivity due to the complexity of the process.

RT2 also used an iterative analytic strategy between April 2018 and September 2018. Three researchers (JK, SCM, AES) provided consensus leading to final results. Each researcher independently mapped CFIR constructs to ORCA items. One research team member (JK) then created a document to display the constructs coded by each researcher for each ORCA item. Items that were in total agreement (all three researchers chose the same construct) were marked green, items with agreement by two researchers were marked yellow, and items with no agreement were marked red. The stoplight document was sent to all research team members for the opportunity to review before reconvening as a group. The team held two phone calls to discuss items marked yellow and red. Each team member provided rationale for the CFIR construct that they selected, and the group discussed until consensus was reached.

Both teams interpreted ORCA items based on each statement’s structure and content, as they would expect naïve lay-(non-research)-responders to interpret its meaning, also guided by available guidance about its intent.

One research team member (JK) compiled the mappings from RT1 and RT2 into a single document. Discrepancies in the mappings were reviewed and discussed among JK, AES, and LJD in March 2019. We used the COREQ (Consolidated criteria for Reporting Qualitative research) to evaluate our work.

## Results

Most ORCA items were mapped to three of the five CFIR domains by both research teams: Inner Setting, Process, and Intervention Characteristics. No items were mapped to the Characteristics of Individuals domain by either team.

Of the 74 ORCA items, the two research teams disagreed 12% of the time (*n* = 9 items) when mapping to the domain level. Of the nine items with disagreement between the two teams, four were mapped to Inner Setting by RT1 but were mapped to Process by RT2, two were mapped to Intervention Characteristics by RT1 but were mapped to Outer Setting by RT2, two were mapped to Process by RT1 but were mapped to Inner Setting by RT2, and one was mapped to Intervention Characteristics by RT1 and mapped to Inner Setting by RT2.

The two research teams disagreed more often (37.8% of the time; *n* = 28 items) when mapping to the level of sub-construct in the CFIR (Additional File 1) because though the teams may have disagreed at the construct level, they may have mapped to another construct within the same domain (e.g., culture vs. learning climate which are both within Inner Setting). The lowest level of disagreement was found for ORCA’s Evidence scale with Context and Facilitation having similar levels of disagreement, indicating the complexity of assessing Context and Facilitation. Mappings for nineteen of the 28 sub-construct disagreements did agree at the domain level.

## Discussion

Independent approaches used by two separate research teams to map items from the ORCA quantitative measurement instrument to CFIR constructs resulted in aligned mapping decisions for close to two thirds of ORCA items. Each team completed this exercise independently, utilizing deep and thoughtful approaches. The mappings were completed in two different periods of time over a 4-year timespan and the teams did not compare decisions until their independent work was completed. Alignment of mapping decisions between the two teams helped bolster the robustness of mappings.

These mappings can be used by researchers for reporting ORCA findings in a way that others, who are using qualitative or other quantitative measures, can use to contrast/compare conceptually related findings from the ORCA. The mappings will also help those using the CFIR as a conceptual framework to identify quantitative measures from the ORCA that could be used for measuring constructs for Inner Setting, Process, and Intervention Characteristics. The ORCA was developed based on the PARiHS framework, which includes 3 domains: Evidence, Context, and Facilitation. The CFIR is broader than the PARiHS, and thus, it is not surprising that ORCA items predominantly mapped to three of the CFIR’s five domains that are most closely related to the PARiHS domains.

The two teams agreed on mappings for two thirds of ORCA items. Lack of agreement for the remaining one third may indicate that the ORCA and CFIR assess different, but complementary, determinants across the domains. Although agreement between research teams at the CFIR domain level of mapping was high (88%), agreement at the more detailed construct level may be more important for practical application, due to constructs providing a more comprehensive evaluation of barrier and facilitators to implementation than the domain level.

The higher rate of disagreement at the construct level may point to insufficient conceptual clarity that contributes to different interpretations of the ORCA and CFIR constructs by different teams and individuals [18]. Conceptual clarity has been identified as a barrier in similar work completed by field experts utilizing concept mapping and Delphi panels to reach consensus [19, 20]. Mapping the ORCA by CFIR domains helps with conceptual clarity because it utilizes the consistent terms and definitions of the CFIR, which is widely used in implementation science. Our mappings (Additional File 1) provide sufficient detail to enable future research teams to replicate our work, which also assists with conceptual clarity. However, the lack of common definitions continues to be a serious problem in using frameworks within implementation research.

Combining the use of the ORCA and the CFIR in studies may allow researchers to gain a more robust picture of the implementation environment, allowing for both depth of information (interviews guided by the CFIR) and breadth of information (surveys using the ORCA). Due to the time-intensive nature of conducting qualitative interviews, researchers can reach a greater number of stakeholders by conducting surveys without losing the depth of knowledge by also completing qualitative interviews. By using both approaches, researchers may also identify determinants of implementation that would not have been gleaned using one approach in isolation. Our mappings (Additional File 1) will help to enrich findings by providing a partial bridge between these two sources of data leading to a more robust triangulation of findings within and across studies.

Though the current work was completed by some of the developers of the ORCA and CFIR, we do not feel that it is necessary for other research teams to have this level of expertise when completing similar mappings. But, it is important that the researchers have a clear understanding of the use and purpose of the instruments and frameworks before starting similar mappings. Researchers need to be mindful to conceptually distinguish concepts prior to completing mappings in order to set up discriminate validation by articulating what a concept is and what it is not. Research teams completing mappings of instruments and frameworks will undoubtedly have some disagreement, but we do not believe this influences the usefulness or replicability of this work. The complexity of completing these types of mappings may be used to identify areas not addressed in existing instruments and where new measures are needed.

Future mappings could include the Tailored Implementation for Chronic Diseases (TICD) framework, which is the most recent consolidated determinants framework and incorporates key aspects of the CFIR and other widely used frameworks and models that could also be mapped to the ORCA [21]. The Organizational Change Manager (OCM), which is a short survey used to detect potential obstacles and improve chances for successful implementation, could be mapped to the CFIR [22]. Taking a different approach, and going in depth into a single construct, Miake-Lye and colleagues have mapped the construct organizational readiness to change, and tools designed to measure it, to the CIFR. This approach of going into more depth in individual constructs is another fruitful approach for future research [23].

A limitation of this work is that both research teams were relatively small, though deeply knowledgeable about the ORCA and/or CFIR. Three of the researchers were involved in the creation of one or both instruments, which may or may not have contributed to differences in coding because of especially deep connection to the respective instrument. Our findings should be viewed as a first attempt to map ORCA items to CFIR constructs which provides a starting point for continued refinement and validation.

## Conclusions

A crosswalk between the CFIR and ORCA has been developed based on mapping decisions from two independent research teams. In addition to Additional File 1, this crosswalk is available online (http://www.cfirguide.org). This crosswalk will help support wider scale comparison and synthesis of findings within and across studies and will assist implementation researchers planning to use the CFIR or the ORCA independently or in combination. Use of both will help strengthen mixed methods approaches widely used in implementation research.

## Supplementary Information

**Additional file 1.** ORCA Mappings completed by RT1 and RT2 by CFIR Domain and Construct.

## Data Availability

Data will be available from the authors on request.

## Abbreviations

CFIR: Consolidated Framework for Implementation Research
EBI: Evidence-based intervention
ORCA: Organizational Resource and Context Assessment
PARIHS: Promoting Action on Research Implementation in Health Services
RCT: Randomized control trial
RT: Research team
