# Correction: Mapping the organizational readiness to change assessment to the Consolidated Framework for Implementation Research

**DOI:** 10.1186/s43058-023-00415-5

**Published:** 2023-03-21

**Authors:** Jennifer Kononowech, Hildi Hagedorn, Carmen Hall, Christian D. Helfrich, Anne C. Lambert-Kerzner, Susan C. Miller, Anne E. Sales, Laura Damschroder

**Affiliations:** 1 Center for Clinical Management Research, VA Ann Arbor Healthcare System, 2800 Plymouth Rd, Ann Arbor, MI USA; 2 Center for Care Delivery and Outcomes Research, Minneapolis VA Health Care System, 1 Veterans Drive, Minneapolis, MN USA; 3 Department of Psychiatry, University of Minnesota School of Medicine, 606 24Th Ave S, Minneapolis, MN USA; 4 Gusek Hall Consulting, 1362 Ryan Ave. W Ste 101, Roseville, MN USA; 5 Seattle-Denver Center of Innovation, VA Puget Sound Health Care System, 1660 S. Columbian Way, Seattle, WA USA; 6 School of Public Health, University of Washington, 1959 NE Pacific St, Seattle, WA USA; 7 Department of Surgery, University of Colorado Anschutz Medical Campus, 12631 East 17Th Avenue, Aurora, CO USA; 8 Department of Health Services, Policy & Practice, Brown University School of Public Health, 121 S. Main Street, Providence, RI USA; 9 Department of Learning Health Sciences, University of Michigan Medical School, 300 N. Ingalls Street, Ann Arbor, MI USA

**Correction: Implement Sci Commun 2, 19 (2021)**

**https://doi.org/10.1186/s43058-021-00121-0**

Following the publication of the original article [1] the authors requested to update the “Competing interests” section as follows:

“The authors declare that Anne Sales is co-Editor-in-Chief of the journal."

## References

[1] Kononowech J (2021). Mapping the organizational readiness to change assessment to the Consolidated Framework for Implementation Research. Implement Sci Commun.

