# Supplementary material for: Mapping the organizational readiness to change assessment to the Consolidated Framework for Implementation Research
Source: Implement Sci Commun. 2021 Feb 13;2:19. doi: 10.1186/s43058-021-00121-0 (PMC7881456; doi:10.1186/s43058-021-00121-0)
Supplement: Supplementary file 1 — Additional file 1. ORCA Mappings completed by RT1 and RT2 by CFIR Domain and Construct. [file 43058_2021_121_MOESM1_ESM.docx]

**Additional File 1:** ORCA Mappings completed by RT1 and RT2 by CFIR Domain and Construct

*Disagreements have been highlighted

| **ORCA Item** | **RT1 Domain** | **RT2 Domain** | **RT1 Construct** | **RT2 Construct** |
| --- | --- | --- | --- | --- |
| Based on your assessment of the evidence basis for this statement, please rate the strength of the evidence in your opinion, on a scale of 1 to 5 where 1 is very weak evidence and 5 is very strong evidence | Intervention Characteristics | Intervention Characteristics | Evidence Strength & Quality | Evidence Strength & Quality |
| Now, please rate the strength of the evidence basis for this statement based on how you think respected clinical experts in {your organization} feel about the strength of the evidence, on a 1 to 5 scale similar to the one above | Intervention Characteristics | Intervention Characteristics | Evidence Strength & Quality | Evidence Strength & Quality |
| **Evidence Scale** |  |  |  |  |
| The {proposed practice changes or guideline implementation}: are (is) supported by randomized control trials (RCTs) or other scientific evidence from the VA | Intervention Characteristics | Intervention Characteristics | Evidence Strength & Quality | Evidence Strength & Quality |
| The {proposed practice changes or guideline implementation}: are (is) supported by randomized control trials (RCTs) or other scientific evidence from other health care systems | Intervention Characteristics | Intervention Characteristics | Evidence Strength & Quality | Evidence Strength & Quality |
| The {proposed practice changes or guideline implementation}: should be effective, based on current scientific knowledge | Intervention Characteristics | Intervention Characteristics | Evidence Strength & Quality | Evidence Strength & Quality |
| The {proposed practice changes or guideline implementation}: are supported by clinical experience with VA patients | Intervention Characteristics | Intervention Characteristics | Evidence Strength & Quality | Intervention Source |
| The {proposed practice changes or guideline implementation}: are supported by clinical experience with patients in other health care systems | Intervention Characteristics | Outer Setting | Evidence Strength & Quality | Patient Needs & Resources |
| The {proposed practice changes or guideline implementation}: conform to the opinions of clinical experts in this setting | Inner Setting | Inner Setting | Compatibility | Compatibility |
| The {proposed practice changes or guideline implementation}: have been well-accepted by VA patients in a pilot study | Intervention Characteristics | Outer Setting | Evidence Strength & Quality | Patient Needs & Resources |
| The {proposed practice changes or guideline implementation}: are consistent with clinical practices that have been accepted by VA patients | Intervention Characteristics | Inner Setting | Evidence Strength & Quality | Compatibility |
| The {proposed practice changes or guideline implementation}: take into consideration the needs and preferences of VA patients | Outer Setting | Outer Setting | Patient Needs & Resources | Patient Needs & Resources |
| The {proposed practice changes or guideline implementation}: appear to have more advantages than disadvantages for VA patients | Intervention Characteristics | Intervention Characteristics | Relative Advantage | Relative Advantage |
| **Context Scale** |  |  |  |  |
| Senior leadership/clinical management in {your organization}: reward clinical innovation and creativity to improve patient care | Inner Setting | Inner Setting | Organizational Incentives & Rewards | Organizational Incentives & Rewards |
| Senior leadership/clinical management in {your organization}: solicit opinions of clinical staff regarding decisions about patient care | Inner Setting | Inner Setting | Networks & Communications | Learning Climate |
| Senior leadership/clinical management in {your organization}: seek ways to improve patient education and increase patient participation in treatment | Outer Setting | Outer Setting | Patient Needs & Resources | Patient Needs & Resources |
| Staff members in {your organization}: have a sense of personal responsibility for improving patient care and outcomes | Inner Setting | Inner Setting | Culture | Relative Priority |
| Staff members in {your organization}: cooperate to maintain and improve effectiveness of patient care | Inner Setting | Inner Setting | Culture | Learning Climate |
| Staff members in {your organization}: are willing to innovate and/or experiment to improve clinical procedures | Inner Setting | Inner Setting | Learning Climate | Culture |
| Staff members in {your organization}: are receptive to change in clinical processes | Inner Setting | Inner Setting | Learning Climate | Culture |
| Senior leadership/Clinical management in {your organization}: provide effective management for continuous improvement of patient care | Inner Setting | Inner Setting | Networks & Communication | Leadership Engagement |
| Senior leadership/Clinical management in {your organization}: clearly define areas of responsibility and authority for clinical managers and staff | Inner Setting | Inner Setting | Structural Characteristics | Goals & Feedback |
| Senior leadership/Clinical management in {your organization}: promote team building to solve clinical care problems | Inner Setting | Inner Setting | Networks & Communications | Learning Climate |
| Senior leadership/Clinical management in {your organization}: promote communication among clinical services and units | Inner Setting | Inner Setting | Networks & Communications | Networks & Communications |
| Senior leadership/Clinical management in {your organization}: provide staff with information on VA performance measures and guidelines | Inner Setting | Inner Setting | Goals & Feedback | Goals & Feedback |
| Senior leadership/Clinical management in {your organization}: establish clear goals for patient care processes and outcomes | Inner Setting | Inner Setting | Goals & Feedback | Goals & Feedback |
| Senior leadership/Clinical management in {your organization}: provide staff members with feedback/data on effects of clinical decisions | Inner Setting | Inner Setting | Goals & Feedback | Goals & Feedback |
| Senior leadership/Clinical management in {your organization}: hold staff members accountable for achieving results | Inner Setting | Inner Setting | Organizational Incentives & Rewards | Organizational Incentives & Rewards |
| Opinion leaders in {your organization}: believe that the current practice patterns can be improved | Inner Setting | Inner Setting | Learning Climate | Learning Climate |
| Opinion leaders in {your organization}: encourage and support changes in practice patterns to improve patient care | Inner Setting | Inner Setting | Learning Climate | Learning Climate |
| Opinion leaders in {your organization}: are willing to try new clinical protocols | Inner Setting | Inner Setting | Learning Climate | Learning Climate |
| Opinion leaders in {your organization}: work cooperatively with senior leadership/clinical management to make appropriate changes | Inner Setting | Process | Learning Climate | Opinion Leaders |
| In general in {your organization}, when there is agreement that change needs to happen: we have the necessary support in terms of budget or financial resources | Inner Setting | Inner Setting | Available Resources | Available Resources |
| In general in {your organization}, when there is agreement that change needs to happen: we have the necessary support in terms of training | Inner Setting | Inner Setting | Access to Knowledge & Information | Available Resources |
| In general in {your organization}, when there is agreement that change needs to happen: we have the necessary support in terms of facilities | Inner Setting | Inner Setting | Available Resources | Available Resources |
| In general in {your organization}, when there is agreement that change needs to happen: we have the necessary support in terms of staffing | Inner Setting | Inner Setting | Available Resources | Available Resources |
| **Facilitation Scale** |  |  |  |  |
| Senior leadership/clinical management will: propose a project that is appropriate and feasible | Process | Process | Planning | Planning |
| Senior leadership/clinical management will: provide clear goals for improvement in patient care | Inner Setting | Inner Setting | Goals & Feedback | Goals & Feedback |
| Senior leadership/clinical management will: establish a project schedule and deliverables | Process | Process | Planning | Planning |
| Senior leadership/clinical management will: designate a clinical champion(s) for the project | Inner Setting | Process | Leadership Engagement | Formally Appointed Internal Implementation Leaders |
| The project clinical champion: accepts responsibility for the success of this project | Process | Process | Champions | Champions |
| The project clinical champion: has the authority to carry out the implementation | Process | Process | Champions | Champions |
| The project clinical champion: is considered a clinical opinion leader | Process | Process | Champions | Champions |
| The project clinical champion: works well with the intervention team and providers | Process | Process | Champions | Champions |
| Senior leadership/clinical management/staff opinion leaders: agree on the goals for this intervention | Inner Setting | Inner Setting | Goals & Feedback | Goals & Feedback |
| Senior leadership/clinical management/staff opinion leaders: will be informed and involved in the intervention | Inner Setting | Inner Setting | Leadership Engagement | Leadership Engagement |
| Senior leadership/clinical management/staff opinion leaders: agree on adequate resources to accomplish the intervention | Inner Setting | Inner Setting | Leadership Engagement | Available Resources |
| Senior leadership/clinical management/staff opinion leaders: set a high priority on the success of the intervention | Inner Setting | Inner Setting | Relative Priority | Relative Priority |
| The implementation team members: share responsibility for the success of this project | Process | Process | Formally Appointed Internal Implementation Leaders | Formally Appointed Internal Implementation Leaders |
| The implementation team members: have clearly defined roles and responsibilities | Process | Process | Formally Appointed Internal Implementation Leaders | Planning |
| The implementation team members: have release time or can accomplish intervention tasks within their regular work load | Inner Setting | Inner Setting | Available Resources | Available Resources |
| The implementation team members: have staff support and other resources required for the project | Inner Setting | Inner Setting | Available Resources | Available Resources |
| The implementation team members: identifies specific roles and responsibilities | Process | Process | Planning | Planning |
| The implementation team members: clearly describes tasks and timelines | Process | Process | Planning | Planning |
| The implementation team members: includes appropriate provider/patient education | Process | Inner | Planning | Access to Knowledge & Information |
| The implementation team members: acknowledges staff input and opinions | Process | Inner | Planning | Learning Climate |
| Communication will be maintained through: regular project meetings with the project champion and team members | Inner Setting | Process | Networks & Communications | Engaging |
| Communication will be maintained through: involvement of quality management staff in project planning and implementation | Inner Setting | Process | Networks & Communications | Engaging |
| Communication will be maintained through: regular feedback to clinical management on progress of project activities and resource needs | Process | Process | Reflecting & Evaluating | Engaging |
| Communication will be maintained through: regular feedback to clinicians on effects of practice changes on patient care/outcomes | Process | Process | Reflecting & Evaluating | Engaging |
| Progress of the project will be measured by: collecting feedback from patients regarding proposed/implemented changes | Process | Process | Reflecting & Evaluating | Reflecting & Evaluating |
| Progress of the project will be measured by: collecting feedback from staff regarding proposed/implemented changes | Process | Process | Reflecting & Evaluating | Reflecting & Evaluating |
| Progress of the project will be measured by: developing and distributing regular performance measures to clinical staff | Process | Process | Reflecting & Evaluating | Reflecting & Evaluating |
| Progress of the project will be measured by: providing a forum for presentation/discussion of results and implications for continued improvements | Process | Process | Reflecting & Evaluating | Reflecting & Evaluating |
| The following are available to make the selected plan work: staff incentives | Inner Setting | Inner Setting | Organizational Incentives & Rewards | Organizational Incentives & Rewards |
| The following are available to make the selected plan work: equipment and materials | Inner Setting | Inner Setting | Available Resources | Available Resources |
| The following are available to make the selected plan work: patient awareness/need | Outer Setting | Outer Setting | Patient Needs & Resources | Patient Needs & Resources |
| The following are available to make the selected plan work: provider buy-in | Process | Process | Engaging | Engaging |
| The following are available to make the selected plan work: intervention team | Process | Process | Formally Appointed Internal Implementation Leaders | Formally Appointed Internal Implementation Leaders |
| The following are available to make the selected plan work: evaluation protocol | Process | Process | Planning | Reflecting & Evaluating |
| Plans for evaluation and improvement of this intervention include: periodic outcome measurement | Process | Process | Planning | Reflecting & Evaluating |
| Plans for evaluation and improvement of this intervention include: staff participation/satisfaction survey | Process | Process | Planning | Reflecting & Evaluating |
| Plans for evaluation and improvement of this intervention include: patient satisfaction survey | Process | Process | Planning | Reflecting & Evaluating |
| Plans for evaluation and improvement of this intervention include: dissemination plan for performance measures | Process | Process | Planning | Reflecting & Evaluating |
| Plans for evaluation and improvement of this intervention include: review of results by clinical leadership | Process | Process | Planning | Reflecting & Evaluating |
